# Disparities in Cognition Among U.S. and Foreign-Born Minority Populations With and Without Diabetes

**DOI:** 10.1093/geroni/igab046.289

**Published:** 2021-12-17

**Authors:** Tiffany Kindratt, Florence Dallo, Laura Zahodne, Kristine Ajrouch

**Affiliations:** 1 University of Texas at Arlington, Arlington, Texas, United States; 2 Department of Public and Environmental Wellness, Rochester, Michigan, United States; 3 University of Michigan, Ann Arbor, Michigan, United States; 4 University of Michigan, Ypsilnati, Michigan, United States

## Abstract

Adults with cognitive limitations and diabetes may be less able to adhere to treatment recommendations. Our aims were to: 1) estimate and compare the prevalence of cognitive limitations and diabetes among foreign-born non-Hispanic whites, blacks, Hispanics, Asians, and Arab Americans to US-born non-Hispanic whites; and 2) examine associations after controlling for covariates. We linked 2002-2016 National Health Interview Survey and 2003-2017 Medical Expenditure Panel Survey data (ages >=45 years, n=122,898). The prevalence of cognitive limitations was highest among foreign-born non-Hispanic whites (9.71%) and Arab Americans (9.40%) and lowest among foreign-born blacks (5.19%). Foreign-born non-Hispanic whites had higher odds (OR=1.36; 95% CI=1.05-1.49) of cognitive limitations than their US-born counterparts. Foreign-born Hispanics with diabetes had greater odds of cognitive limitations (OR=1.91; 95% CI=1.63, 2.24) compared to US-born non-Hispanic whites. Additional findings will be discussed focused on stressors that may contribute to cognition disparities using the immigrant health paradox framework.

